# Dynamics and Time Scales of Higher-Order Correlations in Supercooled Colloidal Systems

**DOI:** 10.1021/acs.jpclett.3c00631

**Published:** 2023-05-12

**Authors:** Nele N. Striker, Irina Lokteva, Michael Dartsch, Francesco Dallari, Claudia Goy, Fabian Westermeier, Verena Markmann, Svenja C. Hövelmann, Gerhard Grübel, Felix Lehmkühler

**Affiliations:** † Deutsches Elektronen-Synchrotron DESY, Notkestraße 85, 22607 Hamburg, Germany; ‡ The Hamburg Centre for Ultrafast Imaging, Luruper Chaussee 149, 22761 Hamburg, Germany; § Institut für Experimentelle und Angewandte Physik, Christian-Albrechts-Universität zu Kiel, Leibnizstraße 19, 24098 Kiel, Germany

## Abstract

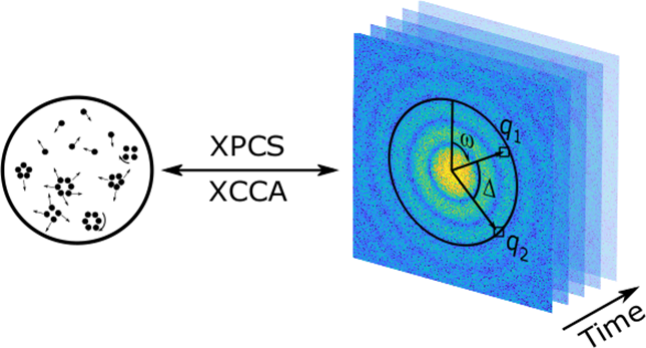

The dynamics and time scales of higher-order correlations are studied in supercooled colloidal systems. A combination of X-ray photon correlation spectroscopy (XPCS) and X-ray cross-correlation analysis (XCCA) shows the typical slowing of the dynamics of a hard sphere system when approaching the glass transition. The time scales of higher-order correlations are probed using a novel time correlation function *g*_*C*_, tracking the time evolution of cross-correlation function *C*. With an increasing volume fraction, the ratio of relaxation times of *g*_*C*_ to the standard individual particle relaxation time obtained by XPCS increases from ∼0.4 to ∼0.9. While a value of ∼0.5 is expected for free diffusion, the increasing values suggest that the local orders within the sample are becoming more long-lived for larger volume fractions. Furthermore, the dynamics of local order is more heterogeneous than the individual particle dynamics. These results indicate that not only the presence but also the lifetime of locally favored structures increases close to the glass transition.

When a supercooled liquid approaches the glass transition, its relaxation time increases by several orders of magnitude while the structure remains liquid-like.^1−5^ Although there has been extensive research into the nature of the glass transition, its mechanisms are mostly unclear. One of the key concepts appears to be dynamical heterogeneity, i.e., the existence of regions with different relaxation times in the system, which occur frequently upon supercooling.^6−9^ This is often discussed together with the appearance of locally favored structures (LFS), such as icosahedral structures forming in this regime.^10−12^

Studies of the structure and dynamics of liquids and glasses show that a local order develops when approaching the glass transition.^13−18^ Many studies investigate these phenomena using colloidal systems, as their structure can be analogous to that in atomic systems and their phase behavior, including approaching the glass transition, can be controlled by different parameters such as volume fraction ϕ.^19^ A recent study showed a link between the number of tetrahedral clusters in hard sphere mixtures and their diffusivity, introducing a way to predict dynamics within the system.^20^ This again demonstrated the important role that the formation of LFS plays in the glass transition. However, although the structural properties of LFS are the subject of many studies, little is known about the time scales and lifetime of LFS and other multiparticle clusters. These can be investigated by higher-order correlations in space and time that, e.g., have been used to reveal a two-step crystallization process in hard sphere colloidal systems via detecting local order.^21−24^ One way to access such higher-order correlations is coherent X-ray scattering.

In general, coherent X-ray scattering methods such as X-ray photon correlation spectroscopy (XPCS)^25−28^ and X-ray cross-correlation analysis (XCCA)^29−31^ are well suited to studying the dynamics and structure of supercooled liquids and glasses because they allow investigation of intrinsic time and length scales, down to molecular dimensions.

Recently, a combined XPCS and XCCA study revealed correlations between the slowing of the dynamics and an increase in structural higher-order correlations preceding the glass transition of colloidal hard spheres.^32^ Because of the increase in the brilliance of current large-scale X-ray sources, new correlation functions can be probed that will enable studies of structure–dynamics correlation over wide time and length scales.^33^ In this work, we use such a newly defined higher-order correlation function combining XPCS and XCCA, which gives access to the lifetime of local order in a colloidal liquid. In the fluid state, the dynamics of the colloids resembles Brownian motion without any influence from higher-order correlations. With an increasing degree of supercooling, the diffusivity measured by XPCS decreases, so that *D*_0_/*D*(*q*_nn_) follows the expected Vogel–Fulcher–Tamann behavior. This is accompanied by a larger increase in the time scales of higher-order correlations by a factor of almost 2. This indicates that higher-order correlations increase as shown previously in refs (13), (32), and (34), and their lifetime increases while approaching the glass transition.

To study the structure and dynamics of supercooled colloidal hard spheres, we performed coherent X-ray scattering experiments at beamline P10, PETRA III (see the Experimental Section for details), for two sample sets of different radii *R* and particle volume fractions ϕ between 0.4 and 0.57. This enables us to access the average static structure as well as dynamics by XPCS and higher-order correlations by XCCA. Both sample sets showed the same behavior with an increasing volume fraction, independent of their individual radii, and will be discussed together below. The static structure of the samples was characterized using structure factors *S*(*q*). The extracted structure factors for some of the samples are shown in Figure 1a as a function of *qR* for the sake of comparison. Modulus of the wave vector *q* is defined as , where λ is the wavelength of the X-rays and θ is the scattering angle. With increasing volume fractions ϕ, the position of the first structure factor peak *q*_nn_, which corresponds to the next-neighbor distance, shifts toward larger *q* values, and its amplitude decreases slightly, indicating a deviation from the Percus–Yevick model. Such a deviation, especially in the peak height, has been observed before in other studies for this range of volume fractions in the vicinity of the glass transition and may indicate that the sample is no longer in a simple liquid state.^35^ As a consequence, *S*(*q*) is no longer suitable to describe the state of the sample, and therefore, information about the dynamics and structural higher-order correlations are needed.

**Figure 1:**
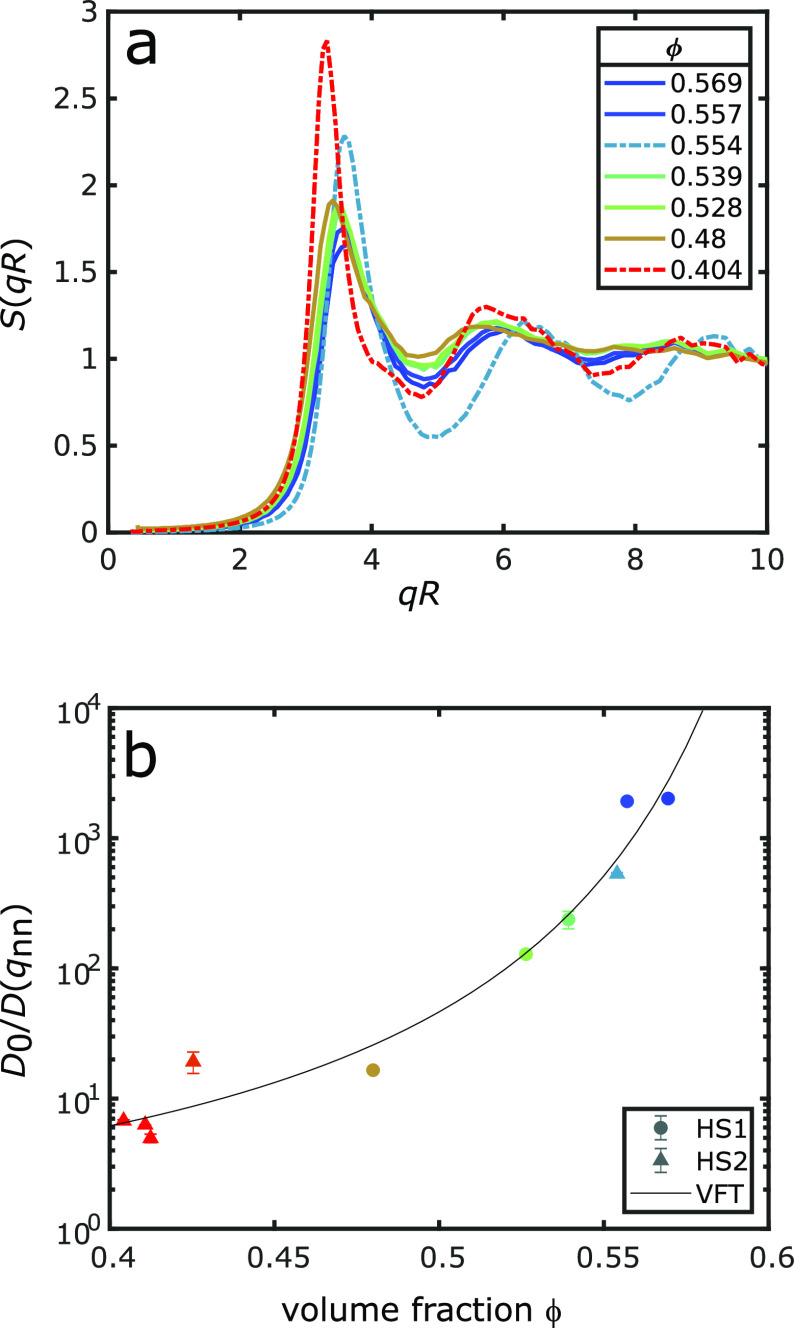
Structure and dynamics. (a) Structure factors *S*(*q*) for samples of HS1 (solid lines) and HS2 (dashed lines), shown as a function of *qR* for *R* of each sample. The corresponding volume fractions ϕ are shown in the legend. For the sake of visibility, not all structure factors are shown. (b) Relative diffusivity *D*_0_/*D*(*q*_nn_) as a function of volume fraction ϕ for HS1 (circles) and HS2 (triangles). The line represents a fit of the VFT law to the data with a ϕ_VFT_ of 0.65 ± 0.04.

The dynamical properties of the samples were studied using XPCS.^25−28^ In XPCS, intensity *I*(*q*, *t*) is correlated via the *g*_2_ function at time *t* and wave vector *q*. The *g*_2_ function is defined as
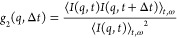
1where ω is the azimuth angle representing all detector pixels within the same *q* ring.

It gives access to intermediate scattering function *f*(*q*, Δ*t*), which can be described by the Kohlrausch–Williams–Watts (KWW) function for many soft matter systems:
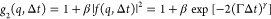
2where Γ is the relaxation rate and γ the KWW exponent. Speckle contrast β depends on the coherence properties of the X-rays and the experimental setup and is 0.4 for this experiment. Characteristic relaxation time τ of the system is directly related to the relaxation rate via τ = 1/Γ and shows a *q* dependence τ ∝ *q*^*p*^ with *p* < 0. Together, exponents γ and *p* specify the dynamics of the system; for example, in the case of free diffusion, γ = 1 and *p* = −2.

Relaxation times τ were determined by fitting eq 2 to the measured *g*_2_ for the probed *q* values. For the sake of comparison of the two sample systems, which have different radii *R*, the relative diffusivity *D*_0_/*D* = *D*_0_*τq*^2^ was calculated. Here, *D*_0_ is the free diffusion coefficient and is given by the Stokes–Einstein representation *D*_0_ = *k*_B_*T*/(6*πηR*), where *k*_B_ is the Boltzmann constant, *T* the temperature, and η the solvent viscosity. The results obtained at the first structure factor peak are shown in Figure 1b. Three different samples of HS2 had a volume fraction (ϕ) very close to 0.4 and showed the same behavior, so only one of the samples is discussed further. Relative diffusivity *D*_0_/*D* increases with volume fraction ϕ. This slowing of the dynamics with an increasing volume fraction is typical for the transition from a liquid to a glassy state and has been observed also for soft colloids.^1,11,36,37^ The increase in *D*_0_/*D* with volume fraction can be modeled empirically by the Vogel–Fulcher–Tammann (VFT) law
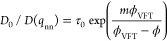
3where τ_0_ is the scaling time, *m* the fragility index, and ϕ_VFT_ the VFT volume fraction, where *D*_0_/*D* → *∞*.^34^ As this VFT model is fully empirical, many approaches have been performed to connect the deceleration to structural properties. For instance, Tong and Tanaka found a VFT relation between the relaxation time and an order parameter of supercooled liquids.^38^ In our previous XCCA study, we could show that structural higher-order correlations represent such an order parameter.^32^ An alternative approach is the recent model of long-lived neighbors.^39,40^ Here, the decrease in the relaxation time could be modeled by observable quantities only, which could be extracted from microscopy experiments on micrometer-sized charged colloids. However, as we do not have access to all of these parameters in the scattering experiment, they cannot be disentangled (see the Supporting Information). A fit of the VFT law to the data is shown in Figure 1b, resulting in a ϕ_VFT_ of 0.65 ± 0.04. This almost matches the random close packing concentration of ϕ_RCP_ = 0.68 for hard spheres with a size dispersity of δ = 15% and has been observed previously.^41−44^

The dynamics is further characterized by exponents γ and *p* (see the Supporting Information). We find three dynamic domains. (i) When ϕ < 0.45, the dynamics characterizes a subdiffusive liquid (*p* ≈ −2, and γ < 1). (ii) When 0.45 < ϕ ≲ 0.55, a transition toward ballistic dynamics (−1.5 < *p* < −1, and γ ≈ 1) is observed. (iii) When ϕ > 0.55, a continued transition toward ballistic and correlated dynamics is observed as found in many colloidal or metallic glasses (γ ≥ 1.5, and −1.5 < *p* < −1). This is often discussed to be a fingerprint of stress-dominated dynamics. Note that in the latter case the relaxation time or diffusivity is expected not to follow the VFT model anymore.^30^ In addition, the dynamics becomes non-isotropically expressed by a direction dependence in the speckle pattern as reported in glasses and gels.^45,46^ However, in the data shown here, the dynamics is isotropic with only very weak modulations with ω (see the Supporting Information), proving that we probe structural dynamics in the sample.

The crossover from the stretched to compressed exponential relaxation, i.e., from γ < 1 to γ > 1, is a current subject of research. It has been found in many different systems undergoing the glass transition, including metallic glasses^47^ and colloidal systems.^32,36,37^ Simulations could connect this transition to changes in local order in metallic glasses.^48^ Furthermore, a recent theory study could model experimental results on the glass transition by connecting stretched exponentials to slowing the dynamics of local relaxation events, while compressed exponentials are related to avalanche-like dynamics in the glass state.^49^ This model reproduced experimental results of metallic glasses well; however, an open question remains whether it can be extended to other glass-forming systems such as colloids, where, e.g., exponents of <0.5 are reported in the supercooled liquid^32,36,37^ (see also the Supporting Information).

The time scales of higher-order correlations were investigated using an approach similar to XPCS. Instead of tracking *I*(*q*, *t*) over time, we defined a correlation function *g*_*C*_(*q*, Δ*t*) that quantifies the time evolution of the cross-correlation function *C*(*q*, Δ). *C*(*q*, Δ) is the convential correlation function used in XCCA and is a measure for higher-order correlations within a sample. It has been successfully used to study orientational order, e.g., local symmetries in colloidal systems^30,32,50,51^ or the structure and in situ self-assembly of colloidal crystals.^52−55^ A recent theoretical study used cross-correlation analysis of XPCS data to determine rotational diffusion coefficients.^33^ Therein, a numerical algorithm for predicting rotational diffusion coefficients has been designed and verified by using simulated data. *C*(*q*, Δ) correlates the scattered intensity at two points separated by angle Δ and is defined as
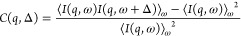
4where ω is the azimuth angle on the detector and *q* = |*q*_1_| = |*q*_2_|. The schematics of the time scales of higher-order correlation analysis along with a simulated scattering pattern and the geometry used in the definition of *C*(*q*, Δ) are shown in Figure 2.

**Figure 2:**
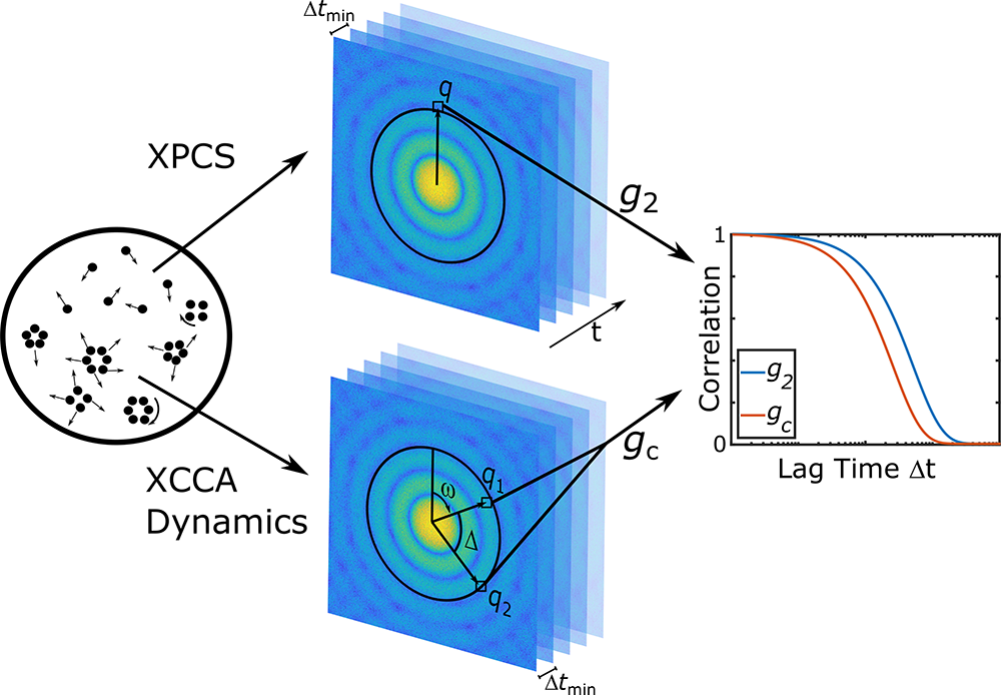
Schematics of the XPCS and time scales of higher-order correlations. A sample is illuminated with coherent X-rays, and the resulting scattering patterns are recorded at time intervals Δ*t*_min_. Via calculation of intensity correlation function *g*_2_ and the time scales of higher-order correlation function *g*_*C*_, more details about the local order, its time scale, and the heterogenity of the system can be obtained.

A correlation function *g*_*C*_(*q*, Δ*t*) that measures the time evolution of *C*(*q*, Δ) is then defined similar to the *g*_2_ function as
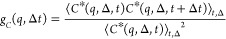
5where C*(*q*, Δ, *t*) = *C*(*q*, Δ, *t*) – min[*C*(*q*, Δ, *t*)], so that C*(*q*, Δ, *t*) ≥ 0 and thus *g*_*C*_(*q*, Δ, *t*) ≥ 0. Compared to *g*_2_, the average in eq 5 is over both *t* and Δ, while it is done over *t* and ω (pixel of the same *q*) in eq 1.

To relate the *g*_*C*_ function to the *g*_2_ function, we performed two simulations: (a) a two-dimensional (2D) simulation for particles undergoing Brownian motion and (b) a 2D simulation of particles in a square symmetry rotating around its center (see the Supporting Information). The first simulation showed that if the sample undergoes free diffusion so that there are no higher-order correlations present,  and as a consequence , i.e., 0.5 for  as in the simulation case. In the second simulation, two cases were investigated: (i) a fixed square symmetry that rotates by a fixed angle around its center per time step and (ii) the particles forming the square being subject to additional Brownian motion in each time step, thus slowly reducing the degree of 4-fold symmetry. The second simulation shows that if orientational order is present and long-lived, *g*_*C*_ shows a much larger characteristic relaxation time. In particular, the square relation  is not valid and the ratio  can become large. Thus, *g*_*C*_ is sensitive to the lifetime of local order independent of dynamics on the single-particle level.

The normalized *g*_*C*_ functions were calculated for each sample and are shown alongside the intermediate scattering functions from the XPCS analysis in Figure 3. The shapes of both correlation functions are very similar for a given sample; both show either a stretched or compressed exponential decay that was modeled by a KWW function. The *g*_*C*_ function typically decreases at slightly shorter times Δ*t* than the intermediate scattering function.

**Figure 3:**
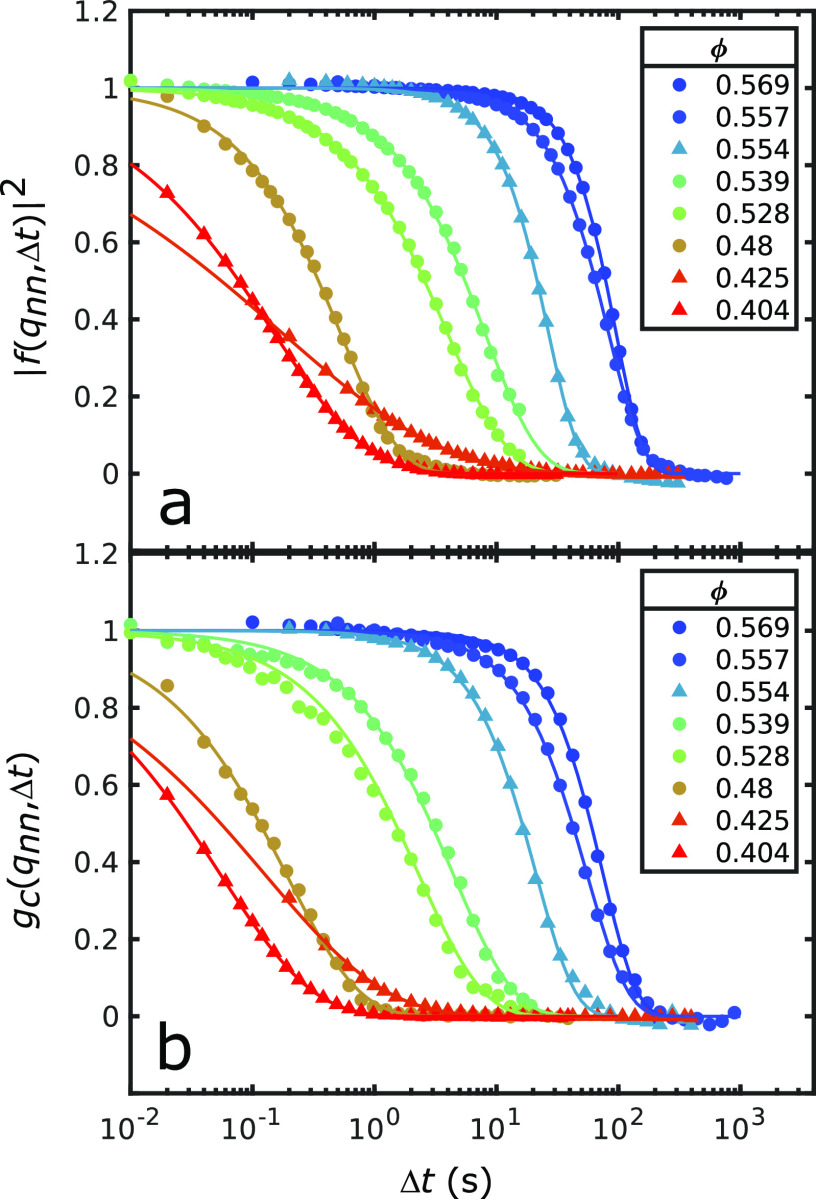
Correlation functions. (a) Intermediate scattering functions and (b) *g*_*C*_ functions at *q*_nn_ for the various volume fractions ϕ of HS1 (circles) and HS2 (triangles). The respective fits are shown as solid lines.

To compare the relaxation times of the *g*_*C*_ and *g*_2_ functions, the quotient of the relaxation times, , was calculated and is shown as a function of volume fraction ϕ in Figure 4a. For the smaller volume fractions,  is between 0.4 and 0.6. This matches the expected results for diffusing particles without a particular structural local order and suggests that the sample is in a liquid state. When ϕ > 0.55, however,  increases from 0.6 to ∼0.9 when ϕ = 0.569. This threshold coincides with the regimes found in the analysis of the dynamics; above ϕ > 0.55, the samples showed signs of ballistic dynamics that are found in many glasses.

**Figure 4:**
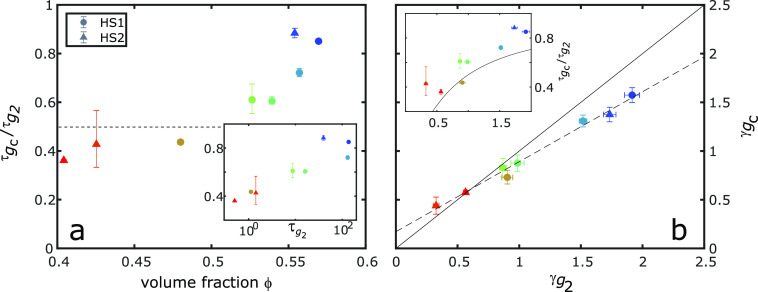
Comparison of the correlation functions. (a) Quotient of the relaxation times, , obtained from the *g*_*C*_ and *g*_2_ functions as a function of volume fraction ϕ. The dashed line signifies . The inset shows  as a function of relaxation time . (b) Exponents  and  plotted against each other. The solid line signifies  and is a guide to the eye, and the dashed line is a linear fit  to the data with *a* = 0.73 and *b* = 0.17. The inset shows  as a function of  with the solid line representing .

The increase in the ratio  indicates that *C*(*q*, Δ) is slowing more quickly than *g*_2_(*q*, Δ*t*) when the volume fraction is increasing. Because *C*(*q*, Δ) is a measure for higher-order correlations that are connected to the local order within the sample, our data indicate that the local order becomes more and more long-lived in the supercooled state. The increase in the ratio of the relaxation times is highlighted even more when  is plotted as a function of , as shown in the inset of Figure 4a. Here, the experimental accuracy of determining ϕ is excluded by focusing on the dynamical properties only. The figure shows the same behavior; the slower the dynamics of the sample, the more long-lived the local order.

As mentioned above, the shape of both functions *g*_2_ and *g*_*C*_ seems to be similar at the same volume fractions. However, taking a closer look, we can find remarkable differences (see Figure 4b). While we find a linear increase in  as a function of , the slope of this linear increase is <1 and  for all cases (except for the smallest volume fraction studied). A smaller value of the KWW exponent γ corresponds to a wider distribution of relaxation times within the exposed sample volume, which can be interpreted as a higher degree of dynamical heterogeneity. Thus, we conclude that the dynamics of the higher-order correlations expressed by *g*_*C*_ are slightly more heterogeneous than the individual particle dynamics, especially in the vicinity of the glass transition. The analysis of two-time correlation functions^25,56,57^ for both XPCS and dynamics of local order shows a small degree of dynamical heterogeneity, but a direct comparison between both is limited by different statistical accuracies (see the Supporting Information).

The inset of Figure 4b shows the ratio  as a function of  with the solid line representing the behavior expected for  of . The lower values of  that represent the samples at small volume fractions follow this expected behavior for (sub)diffusion. The higher values deviate from this diffusion expectation. Instead,  is larger and thus well above the solid line in the inset of Figure 4b and increases further with . This indicates that the *g*_*C*_ values of these samples cannot be explained by the diffusion relation  and in fact exhibit long-lived local order.

In conclusion, we studied the dynamics and time scales of higher-order correlations in hard sphere systems using a novel combination of spatial and temporal correlation functions. By combining the correlation functions from XCCA and XPCS, we were able to gain a unique insight into the correlation between structure and dynamics in colloidal systems. For samples with a volume fraction above ϕ ≈ 0.55, we found that the ratio  increases to 0.9. These values are significantly higher than the values for diffusive samples without any preferred local order of , evidencing that the local order becomes more long-lived when the glass transition is approached. Furthermore, the dynamics of local order are slightly more heterogeneous than the individual particle dynamics.

Our results are fundamental for understanding supercooled colloidal fluids in the vicinity of the glass transition. The experimental approach not only shows that local structures evolve but also provides access to their lifetime. This could help in understanding dynamical heterogeneities and the formation of locally favored structures. Additionally, as our approach combines studies of structure and dynamics using coherent X-rays, it will benefit from diffraction-limited storage rings (DLSR) such as PETRA IV, ESRF-EBS, or APS-U.^58−61^ Most importantly, our approach is not limited to studying correlations in colloidal systems. Because of the short wavelength of X-rays, it can be extended to detect structure–dynamics correlation down to molecular length scales and will thus be a valuable tool for studies of glass and phase transitions of different materials. Furthermore, it may provide access to crystallization kinetics, e.g., during self-assembly of nanocrystals,^55^ or detect transient structures in molecular liquids such as water.

## Experimental Section

We performed X-ray scattering experiments on hard sphere poly(methyl methacrylate) (PMMA) particles dispersed in decalin at beamline P10 of PETRA III, DESY. This sample system is known to be an excellent experimental representation of a hard sphere system.^62^ Two samples, HS1 with *R* = 94 ± 10 nm and a size dispersity of δ = 17% and HS2 with radius *R* = 73 ± 3 nm and δ = 10%, were investigated. To study supercooled liquids, highly concentrated PMMA stock samples were diluted with known quantities of decalin to produce volume fractions (ϕ) ranging from 0.4 to 0.57. The samples were studied in ultra-small-angle X-ray scattering (USAXS) geometry with a beam size of 30 μm × 30 μm and a photon energy of 8.5 keV, corresponding to a wavelength of 1.46 Å. An Eiger X 4M instrument was used as a detector with a sample–detector distance of 21.2 m. The volume fractions were determined by modeling the static structure factors with the Percus–Yevick model, also taking into account the size dispersity of the particles.^63,64^ The absolute error of the determined volume fractions is estimated to be ≲3%.^65^ Note that the relative error between different concentrations for each sample is much smaller because they were obtained by dilution from the same stock solution.

## Supporting Information Available

2D simulations for particles undergoing free diffusion and additional analysis of particle dynamics (PDF)
