# Supplementary material for: Dynamics and Time Scales of Higher-Order Correlations in Supercooled Colloidal Systems
Source: J Phys Chem Lett. 2023 May 12;14(20):4719–25. doi: 10.1021/acs.jpclett.3c00631 (PMC10226114; doi:10.1021/acs.jpclett.3c00631)
Supplement: Supplementary file 1 — jz3c00631_si_001.pdf [file jz3c00631_si_001.pdf]

Supporting Information: Dynamics and Timescales of Higher Order Correlations in Supercooled Colloidal Systems

Nele N. Striker,*,† Irina Lokteva,†,‡ Michael Dartsch,†,‡ Francesco Dallari,†,§ Claudia Goy,† Fabian Westermeier,† Verena Markmann,†,∥ Svenja C. Hövelmann,†,¶ Gerhard Grübel,†,‡,⊥ and Felix Lehmkühler†,‡

†*Deutsches Elektronen-Synchrotron DESY, Notkestr. 85, 22607 Hamburg, Germany*

‡*The Hamburg Centre for Ultrafast Imaging, Luruper Chaussee 149, 22761 Hamburg, Germany*

¶*Institut für Experimentelle und Angewandte Physik, Christian-Albrechts-Universität zu Kiel, Leibnizstraße 19, 24098 Kiel, Germany*

§*Current address: Dipartimento di Fisica e Astronomia, "Galileo Galilei," Università degli Studi di Padova, Via F. Marzolo 8, 35131 Padova, Italy*

∥*Current address: Department of Physics, Technical University of Denmark, Kgs. Lyngby 2800, Denmark*

⊥*Current address: European X-ray Free-Electron Laser, Holzkoppel 4, 22869 Schenefeld, Germany*

E-mail: nele.striker@desy.de

2D Simulations

Before analysing the timescales of higher order correlation on experimental data, it was first demonstrated on simulated Brownian motion of 2D disks with no particle-particle interaction.

For a total of 100 particles, Brownian motion was simulated by moving each particle in random directions. The step size of the movement follows a Gaussian distribution and was chosen so that a decorrelation of the g_2 function can be observed within 1000 steps. The path of one particle over 1000 steps is shown in Figure S1.

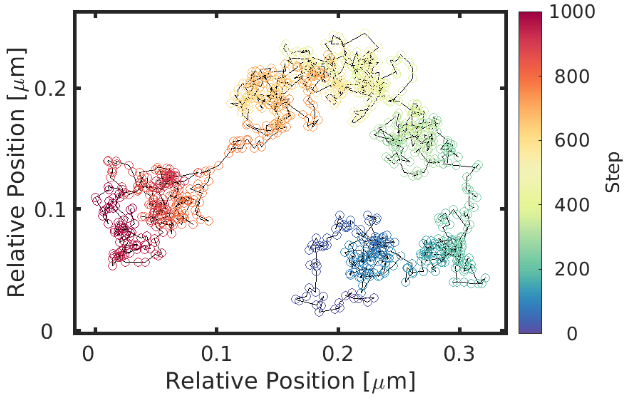

Figure S1: Path of a simulated particle over 1000 steps.

Both, the normalized g_2 and the normalized g_C functions at various q-values were calculated for this system and are shown in Figure S2. The q-values were defined by assuming a wavelength of $\lambda = 1.5$, a distance between the sample and detector of $d = 5$m and a detector pixel size of 50×50 µm^2. A q-map was then calculated via $q = \frac{4\pi}{\lambda} \sin(\arctan(\frac{r}{d})/2)$, where r is the distance from the center. The g_C functions decorrelate from 1 to 0 over a time Δt and have a very similar shape when compared to the g_2 functions. For a given value of q the g_C functions always decorrelate faster than the g_2 functions. Eq. [2] of the main manuscript was fitted to both the g_2 and the g_C functions and their relaxations times τ were determined.

The quotient of the relaxation times τ_{g_C}/τ_{g_2} is shown in Figure S3 as a function of q. For most q, τ_{g_C}/τ_{g_2} is around 0.5. At $q = 0.05$ nm^{-1}, τ_{g_C}/τ_{g_2} decreases significantly, with a much larger errorbar. This is due to low statistics at this q, since it corresponds to the first minimum of the intensity due to the single particle form factor. This shows, that if the sample shows only diffusion and no spatial correlations, $g_C(\Delta t)$ decrease twice as fast as $g_2(\Delta t)$, $\tau_{g_C} = 0.5\tau_{g_2}$. Note that this

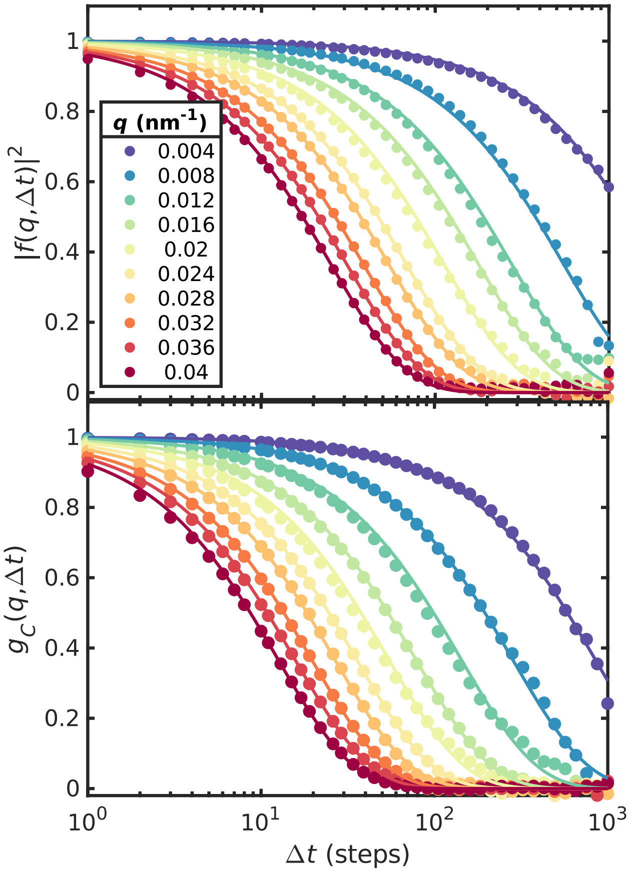

Figure S2: Intermediate scattering functions $|f(q,\Delta t)|^2$ and normalized $g_C(q,\Delta t)$ functions for various values of q. The corresponding fits are shown as solid lines.

result agrees with the fact, that for g_C squares of the intensity I are correlated whereas the intensity I is directly correlated for g_2.

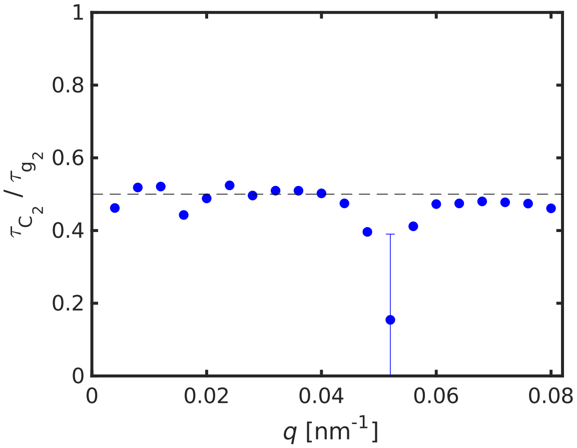

Figure S3: Ratio τ_{g_C}/τ_{g_2} for the simulated particles.

As an example of a non diffusive system, a system showing a square symmetry that rotates around its center was simulated. The g_2 and g_C functions for this example are shown in Figure S4 as dashed lines. In this case, the g2 function shows the typical oscillatory shape that has been reported in many studies (e.g. on flowing liquids and soft matter under (oscillatory) shear). As such a rotation does not alter the higher-order correlation and thus $C(\Delta)$, its time correlation is constant, indicating a stable structural correlation. In a second step, diffusive motion was added to the individual particles while the rotation of the whole structure was still present. This means, that the higher-order correlation vanishes with time, as can be seen by the drop of g_c with increasing rotation angle in Figure S4.

Additional analysis of the dynamics

The determined relaxation times τ are shown as a function of q in Figure S5. For all samples τ decreases with increasing q. For volume fractions $\phi < 0.45$ a slowing down of the dynamics at $q_{nn} = 0.046$ nm^{-1} is observed, which is the position of the first structure factor peak and is known as the de Gennes narrowing. Functions of type $\tau = aq^p$ were fitted to the measured values and

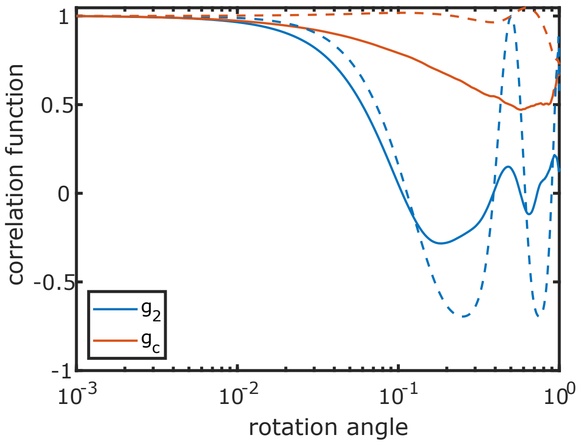

Figure S4: Correlation functions g_2 and g_C for a simulated square rotating around its center without diffusive motion of the individual particles (dashed lines) and with diffusive motion of the individual particles (solid lines).

are shown as solid lines in Figure S5. For HS2, the functions were only fitted to the values of $q < 0.04$ nm, that did not show the de Gennes narrowing.

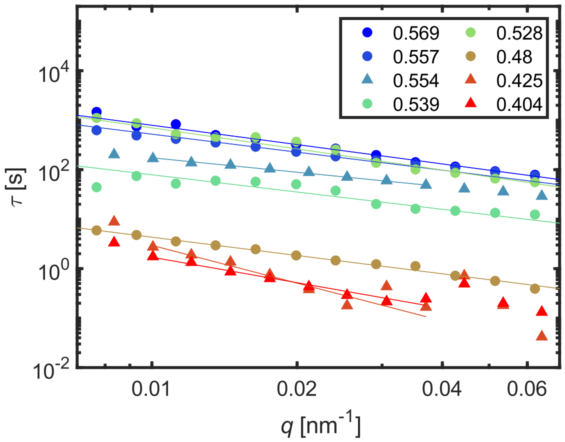

Figure S5: Relaxation times τ as a function of q for HS1 (circles) and HS2 (triangles) at various volume fractions.

The KWW exponents γ, determined by fitting Eq. 2 to the measured g_2, are shown in Figure S6, along with the exponents p of the q-dependence of the relaxation time. Together, the KWW exponent γ and the exponent p specify the dynamics of a system. We find three dynamic domains: (i) for $\phi < 0.45$ the dynamics characterize a subdiffusive liquid ($p \approx -2$ and $\gamma < 1$), (ii) for $0.45 < \phi \lesssim 0.55$ a transition towards ballistic dynamics ($-1.5 < p < -1$ and $\gamma \approx 1$) is ob-

served, and (iii) for $\phi > 0.55$ stress-dominated dynamics are found as in many (colloidal) glasses ($\gamma >= 1.5$ and $-1.5 < p < -1$). The boundaries of these domains, however, are not clear-cut.

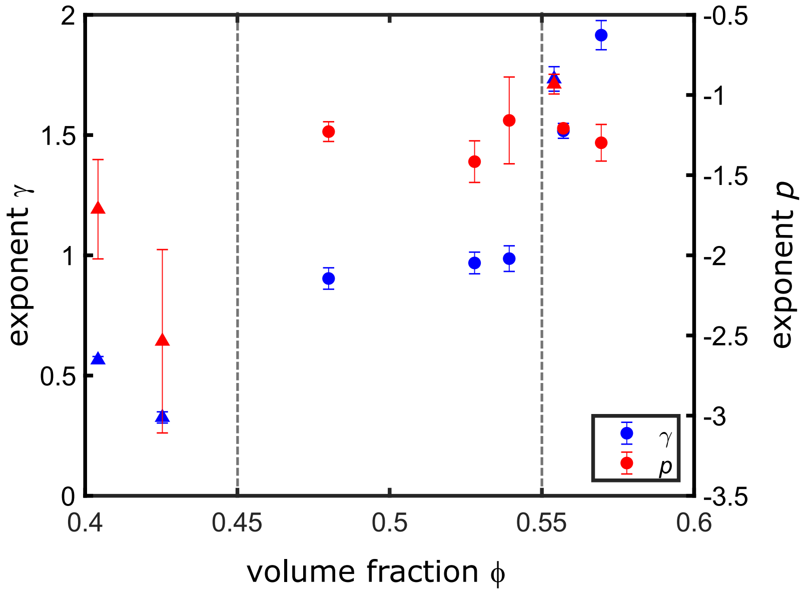

Figure S6: Exponents γ and p as a function of the volume fraction ϕ for HS1 (circles) and HS2 (triangles). The grey, dashed lines indicate the dynamical regions that we found.

In order to check for non-isotropic dynamics that are found for stress-dominated dynamics, g_2 were calculated as a function of the azimuth angle ω. The relaxation times as a function of ω are shown in Fig S7 for three different volume fractions. They show no significant change in behaviour for different angles ω.

Based on the model introduced in S1 we used the function $D_0/D(q_{nn}) = D \exp\left(a \cdot \phi^{1+b}\right)$ with three fit parameters D, a and b to fit our data to the model of long-lived neighbours. Note that this model relies on parameters that could be measured experimentally in the microscopy experiment (e.g. number of neighbours, $g(r)$, spring constant k) but cannot be obtained in our scattering experiment on smaller particles. Therefore we cannot further disentangle our fitting parameter a. The fits to the data are shown in Figure S8 with the black line representing the fit to the VFT model. In Fit 2 all three parameters are fitted, while Fit 3 sets $D = 1$ as expected in the dilute limit. For

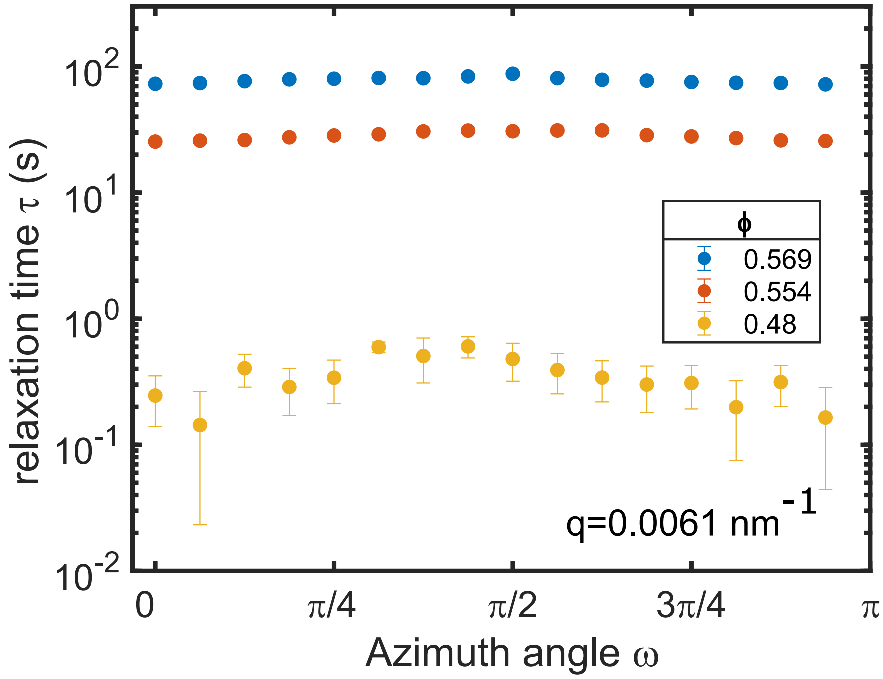

Figure S7: Relaxation times τ as a function of the azimuth angle ω at $q = 0.0061$ nm^{-1}.

b we find rather large values of $b = 7.4$ for Fit 2 and $b = 5.0$ for Fit 3. Note that in the model of long-lived neighours also the pre-factor a changes with ϕ, so our fit here can only be understood as a rough estimate.

We calculated two-time correlation functions to check our samples for dynamical heterogeneity.[S2–S5] They are shown in Fig. S9 for the two highest volume fractions and for the g_2 and the g_C functions. The two-time correlation functions show stable dynamics with only small modulations. From the two-time correlations we determined the variance χ which is shown in Fig.S10 for the g_2 functions. The order of magnitude of χ is 10^{-4}, which is low compared to other heterogeneous systems where variances of at least 10^{-3} to 10^{-1} are reported.[S6–S10] Due to low statistics χ could not be calculated for the g_C functions. Overall, this showed that the degree of dynamical heterogeneity is small.

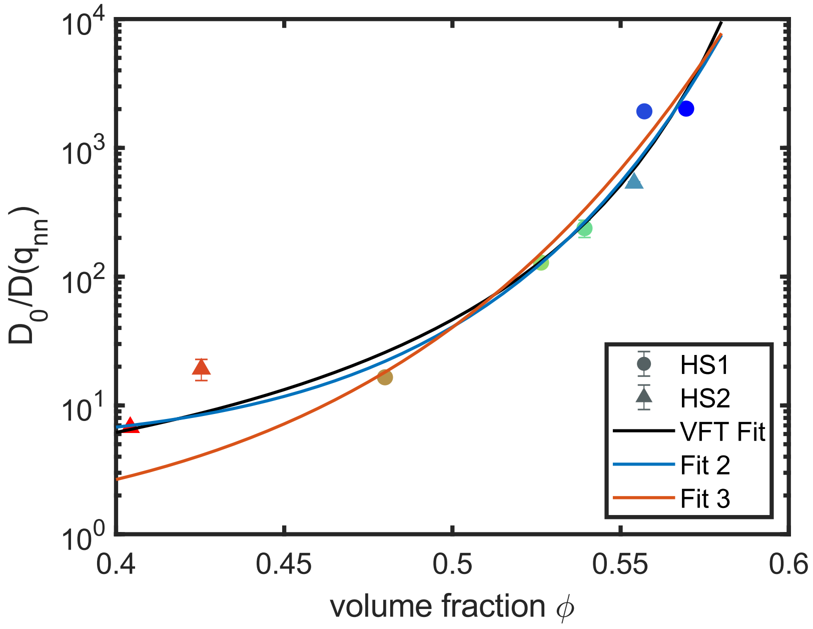

Figure S8: Relative diffusivity $D_0/D(q_{nn})$ as a function of the volume fraction ϕ for HS1 (circles) and HS2 (triangles). The lines represent fits to different models.

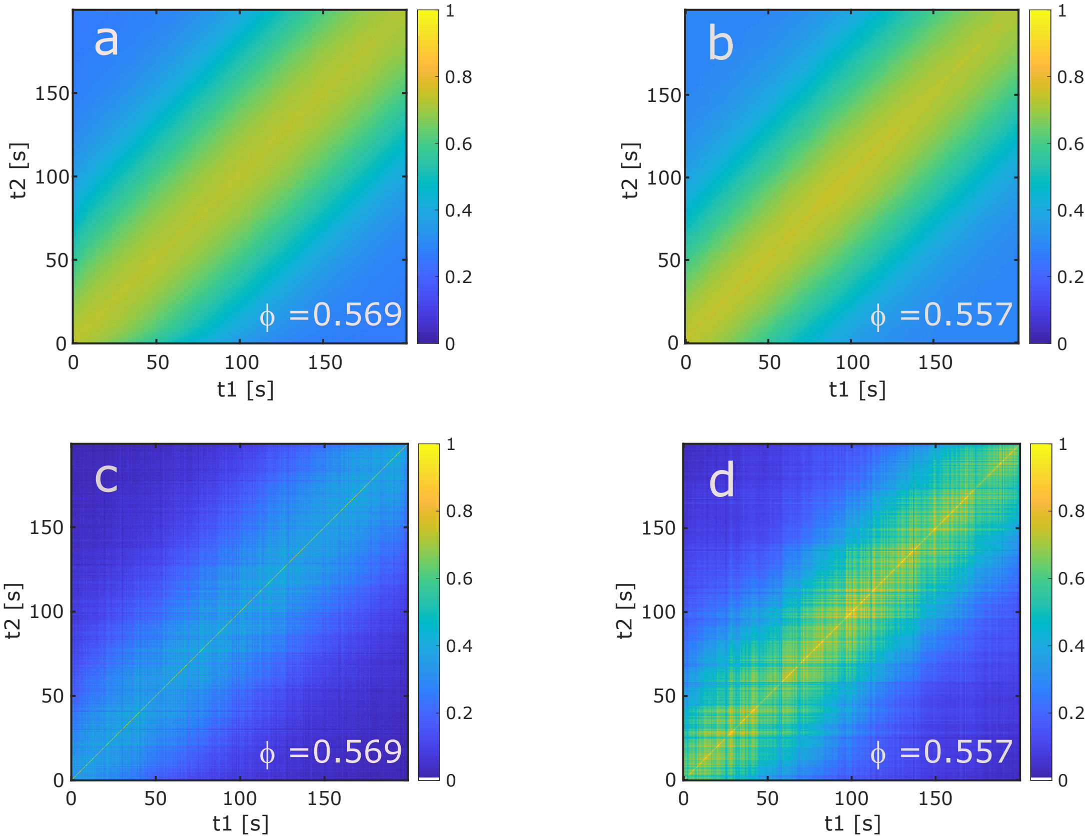

Figure S9: Two-time correlation functions at $q = q_{nn}$ for (a),(b) the g_2 and (c), (d) the g_C functions.

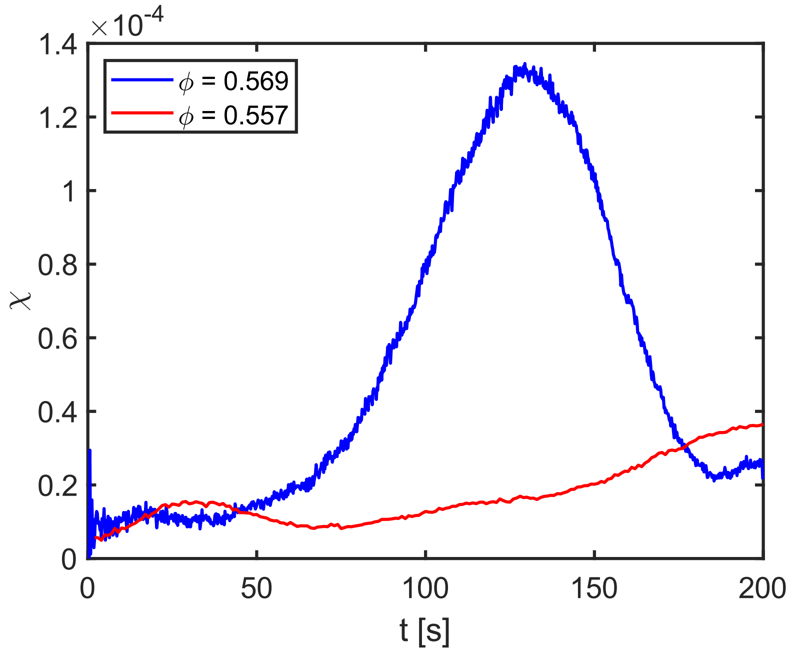

Figure S10: χ of the g_2 functions.

References

(S1) Higler, R.; Krausser, J.; van der Gucht, J.; Zaccone, A.; Sprakel, J. Linking slow dynamics and microscopic connectivity in dense suspensions of charged colloids. *Soft Matter* **2018**, *14*, 780–788.

(S2) Lehmkühler, F.; Roseker, W.; Grübel, G. From femtoseconds to hours—measuring dynamics over 18 orders of magnitude with coherent X-rays. *Appl. Sci.* **2021**, *11*, 6179.

(S3) Madsen, A.; Leheny, R. L.; Guo, H.; Sprung, M.; Czakkel, O. Beyond simple exponential correlation functions and equilibrium dynamics in x-ray photon correlation spectroscopy. *New J. Phys.* **2010**, *12*, 055001.

(S4) Bikondoa, O. On the use of two-time correlation functions for X-ray photon correlation spectroscopy data analysis. *J.Appl. Cryst.* **2017**, *50*, 357–368.

(S5) Duri, A.; Bissig, H.; Trappe, V.; Cipelletti, L. Time-resolved-correlation measurements of temporally heterogeneous dynamics. *Phys. Rev. E* **2005**, *72*, 051401.

(S6) Conrad, H.; Lehmkühler, F.; Fischer, B.; Westermeier, F.; Schroer, M. A.; Chushkin, Y.; Gutt, C.; Sprung, M.; Grübel, G. Correlated heterogeneous dynamics in glass-forming polymers. *Phys. Rev. E* **2015**, *91*, 042309.

(S7) Dallari, F.; Martinelli, A.; Caporaletti, F.; Sprung, M.; Baldi, G.; Monaco, G. Stochastic atomic acceleration during the X-ray-induced fluidization of a silica glass. *PNAS* **2023**, *120*, e2213182120.

(S8) Jain, A.; Schulz, F.; Lokteva, I.; Frenzel, L.; Grübel, G.; Lehmkühler, F. Anisotropic and heterogeneous dynamics in an aging colloidal gel. *Soft Matter* **2020**, *16*, 2864–2872.

(S9) Perakis, F.; Amann-Winkel, K.; Lehmkühler, F.; Sprung, M.; Mariedahl, D.; Sellberg, J. A.; Pathak, H.; Späh, A.; Cavalca, F.; Schlesinger, D.; Ricci, A.; Jain, A.; Massani, B.; Aubree, F.; Benmore, C. J.; Loerting, T.; Grübel, G.; Pettersson, L. G. M.; Nilsson, A.

Diffusive dynamics during the high-to-low density transition in amorphous ice. *PNAS* **2017**, *114*, 8193–8198.

(S10) Begam, N.; Ragulskaya, A.; Girelli, A.; Rahmann, H.; Chandran, S.; Westermeier, F.; Reiser, M.; Sprung, M.; Zhang, F.; Gutt, C.; Schreiber, F. Kinetics of network formation and heterogeneous dynamics of an egg white gel revealed by coherent X-ray scattering. *Phys. Rev. Lett.* **2021**, *126*, 098001.
